# Optimizing High-Volume Fly Ash Mortar with Nano-SiO_2_ and PVA Fibers: Performance and Microstructure

**DOI:** 10.3390/nano15110837

**Published:** 2025-05-30

**Authors:** Junliang Zhao, Zhongkun Wang, Gengying Li, Shengliang Lu

**Affiliations:** 1 College of Civil Engineering and Architecture, Wenzhou University, Wenzhou 325035, China; zhaojunliang@wzu.edu.cn (J.Z.); wangzhongkun@wzu.edu.cn (Z.W.); 2 Key Laboratory of Engineering and Technology for Soft Soil Foundation and Tideland Reclamation of Zhejiang Province, Wenzhou 325035, China; 3 College of Water Conservancy and Civil Engineering, South China Agricultural University, Guangzhou 510642, China; ligengying@scau.edu.cn; 4 School of Civil Engineering and Architecture, Wenzhou Polytechnic, Wenzhou 325035, China

**Keywords:** nano-SiO_2_, PVA fiber, mechanical properties, drying shrinkage, high-volume fly ash mortar

## Abstract

The mechanical properties, capillary water absorption, drying shrinkage, and morphology of high-volume fly ash mortar were investigated. The mortar contained 0~2.5 wt. % nano-silicon dioxide (nano-SiO_2_, NS) and 0~1.5 vol.% polyvinyl alcohol (PVA) fiber, with fly ash (FA) replacing 50% of cement by weight. The experimental results demonstrated that the synergistic incorporation of NS and PVA fiber significantly improved the mortar’s mechanical performance. At 7 days of age, the flexural and compressive strength of mortar containing 1.5% NS and 1.0% PVA fiber increased by 105.8% and 25.1%, respectively, compared to the control mortar (without NS or PVA fiber). Moreover, NS and PVA fiber significantly reduced the capillary absorption rate and drying shrinkage. The composite addition of 2.0% NS and 1.0% PVA fiber led to a notable reduction in drying shrinkage: at 7, 14, 28, 90, and 180 days, the drying shrinkage decreased by 38.3%, 33.3%, 30.0%, 31.6%, and 31.4%, respectively, relative to the control mortar. The scanning electron microscopy and mercury intrusion porosimetry results indicated that NS and PVA fibers effectively improved the micropore structure of the mortar.

## 1. Introduction

Fly ash (FA), a major solid waste, poses adverse effects on land and water resources due to its long-term accumulation. Finding a reasonable way to reutilize this industrial byproduct has become an urgent issue. As a major player in the construction industry, China relies heavily on cement, which has an irreplaceable position among building materials. However, the production process of cement releases a large amount of carbon dioxide, accounting for 7–9% of global CO_2_ emissions [1,2,3]. Currently, emissions from cement plants alone contribute to over 5% of global CO_2_ emissions [4]. In response to the national “carbon peak, carbon neutrality” policy and to increase the utilization of industrial waste, many scholars have begun focusing on green concrete. Researchers have initiated studies on low-carbon materials and CO_2_ emission reduction through carbonation reactions [5,6]. The high-volume fly ash concrete has thus emerged. This type of concrete aims to reduce dependence on cement and achieve environmentally friendly production. Fly ash itself has several advantages as a cementitious material, such as its small diameter helping to fill voids to increase density, pozzolanic activity enhancing macroscopic performance, and spherical structure providing a ball-bearing effect to improve the workability of concrete [7,8,9,10,11,12,13,14]. However, fly ash also has drawbacks, as its molecules remain inactive during the initial hydration process, leading to a lower degree of early hydration reactions and affecting early strength [15].

Nano-silica (nano-SiO_2_, NS) possesses a higher specific surface area, endowing it with a significant reactivity enhancement in the initial hydration stages [16,17,18], and can effectively activate fly ash during early hydration [19]. Small dosages of NS can effectively enhance the early strength of fly ash concrete. For example, the addition of 2% NS can increase the three-day compressive strength of high-volume fly ash concrete by 21.3% [20]. This can be explained because NS can promote the hydration process to consume C_3_S and C_2_S to form more C-S-H gels and reduce the content of Ca(OH)_2_ [21,22,23,24]. Although studies have extensively investigated the mechanical properties [25,26,27,28,29,30] and durability [31,32,33,34] of NS-containing concrete, research on the drying shrinkage performance of fly ash concrete—particularly high-volume fly ash concrete modified with NS—remains limited.

Fly ash concrete contains numerous micropores and microcracks. Fluctuations in internal moisture due to environmental humidity variations induce shrinkage, deformation, and cracking. These cracks elevate risks of carbonation, freeze–thaw damage, and steel corrosion, compromising structural safety, reliability, and service life. The drying shrinkage of concrete is mainly related to the physical adsorption of water by cement stone, C-S-H, and the migration amount and rate of water adsorbed by capillary pores. Usually, the more pores and microcracks in concrete, the greater the migration amount and rate of adsorbed water. Due to the ability of fibers to suppress the formation of cracks, the drying shrinkage of concrete can be reduced by introducing fibers into the concrete [35]. Generally, the higher the elastic modulus and the better the interfacial adhesion between the fibers and cement, the more significant the inhibitory effect [36,37]. PVA fiber has a high elastic modulus and tensile strength, and also has a good bonding performance with cement [38,39,40,41,42]. Incorporating PVA fiber into cementitious materials effectively enhances toughness, limits crack propagation, and improves the mechanical properties and durability of composite materials [43,44,45,46]. While PVA fiber-reinforced concrete has been widely studied for mechanical performance [47,48] and crack resistance [49,50], research on its shrinkage behavior in high-volume fly ash concrete remains scarce.

In this paper, the effects of NS and PVA fiber on the mechanical properties, capillary absorption, and drying shrinkage of mortar with high-volume fly ash are studied, and the mechanism of action is analyzed by microscopic scanning electron microscopy.

## 2. Materials and Methods

### 2.1. Materials

The P.O. 42.5-grade ordinary Portland cement (Wenzhou Conch Cement Co., Ltd., Wenzhou, China) employed in this investigation was sourced from a regional producer. Its key physicochemical properties are systematically presented in Table 1. Grade II fly ash (Wenzhou Building Materials Company, Wenzhou, China) was used to partially substitute cement. Tap water was used as the mixing water, while standard sand (ISO) served as the fine aggregate. NS (Hebei Keze Metal Materials Co., Ltd., Shijiazhuang, China) was selected as the mineral additive, with its essential characteristics listed in Table 2. Additionally, the key properties of the PVA fibers (Japan Co., Ltd., Tokyo, Japan) are presented in Table 3. To enhance the workability of the cement mortar, a polycarboxylic acid superplasticizer was incorporated in this study.

### 2.2. Mix Design and Test Methods

Table 4 summarizes the fifteen distinct mixture formulations examined in this experimental program. The fly ash dosage was maintained at 50% cement replacement by weight across all mixes. The control mix (designated ‘FA’) contained neither NS nor PVA fibers. The other fourteen experimental mixes incorporated varying proportions of NS, PVA fibers, and both additives. The volumetric content of PVA fiber ranged from 0.2% to 1.5%, while the weight content of NS varied between 0.5% and 2.5%. Each mixture was assigned a self-explanatory name, incorporating the additive type (NS or PVA) and its corresponding content. The compressive strength, flexural strength, capillary water absorption, and drying shrinkage of each mixture were tested following the methods detailed in the subsequent subsections.

#### 2.2.1. Test Methods of Mechanical Properties

**Figure 1:**
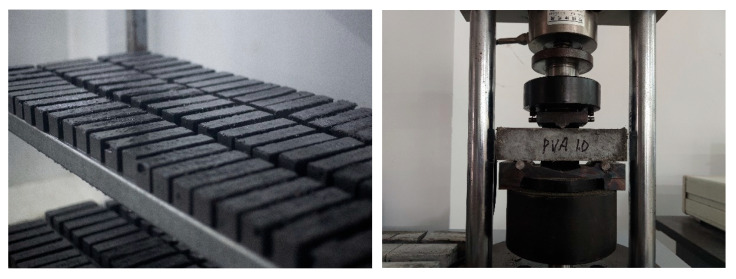
Specimens and mechanical strength test.

The experimental program utilized 40 × 40 × 160 mm prismatic specimens (Figure 1) for mechanical property evaluation. Following standard curing room conditioning, specimens underwent strength testing at 7-day and 28-day intervals using computer-controlled hydraulic equipment compliant with GB/T 17671-2021 [51]. Flexural strength was measured at 50 N/s with automatic load recording, while compressive tests employed 2.4 kN/s loading under instrument-controlled conditions.

#### 2.2.2. Capillary Absorption Test Method

**Figure 2:**
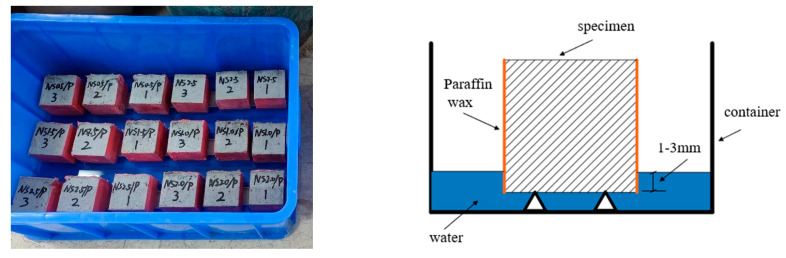
Specimens and schematic diagram of capillary water absorption test device.

The capillary absorption test in this study adhered to the specifications of ASTM C1585-13. Standard cubic specimens (Figure 2) with dimensions of 70.7 mm × 70.7 mm × 70.7 mm were used, and the test was conducted in an environment with a controlled temperature of 20 ± 2 °C. After curing in a standard curing room for 28 days, the specimens were placed in an oven at 80 ± 5 °C for 48 h. Upon removal from the oven, prepared paraffin wax was heated to a liquid state and evenly applied to all four side-surfaces of the cubes. This ensured one-dimensional capillary absorption during the test and prevented water ingress from the sides. After sealing the specimen, it was allowed to stand for 20 min until the wax was completely solidified. The weight of the specimen was measured as $m_{0}$ using an electronic scale. The experimental setup consisted of a support platform placed at the bottom of a container. Tap water was added until the liquid level reached 1–3 mm above the platform’s upper surface. The specimen was then carefully positioned with its testing surface in contact with the platform, and the timer was immediately started (Figure 1). Capillary absorption measurements were recorded at 5, 10, 20, 30, 60, 120, 180, 360, and 720 min. At each interval, the specimen was removed, gently wiped with a soft cloth, inverted, and weighed, yielding $m_{n}$. The capillary absorption was calculated using Equation (1):

(1) $$I_{n}=\frac{m_{n}-m_{0}}{A}$$

where $I_{n}$ is the capillary absorption expressed in millimeters (g/mm^2^); $m_{0}$ is the mass of the dried specimen before testing; $m_{n}$ is the mass of the specimen after water absorption, where *n* = 1, 2, 3, …; and A is the contact surface area between the specimen and water (m^2^). The arithmetic average of the values obtained from three specimens were taken as the capillary absorption, and the result was recorded with a precision up to 0.1 g.

#### 2.2.3. Drying Shrinkage Test Method

**Figure 3:**
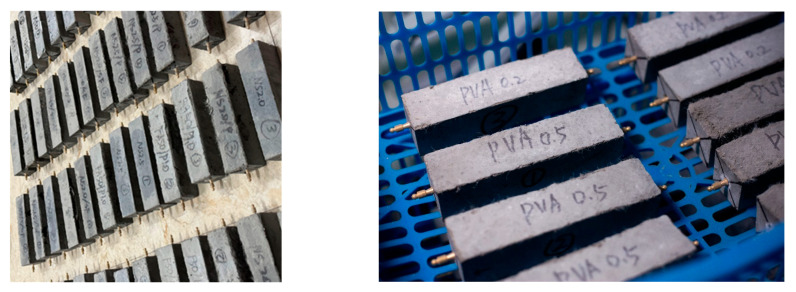
Specimens of drying shrinkage.

The drying shrinkage test was performed in accordance with the Chinese code (ISO 679:2009) [52]. Mortar specimens were cast in 40 mm × 40 mm × 160 mm molds and stored at 20 ± 2 °C for 48 h before demolding. The testing direction was marked and numbered. Two copper nails were adhered to the center of each end face. After 4 h of curing, the initial length was measured using an electronic vernier caliper along the designated testing direction. Subsequently, the specimens were transferred to a controlled environment (20 ± 2 °C, 60 ± 5% relative humidity). Length measurements were taken at 1, 3, 7, 14, 28, 90, 180, and 270 days, recorded as the natural drying lengths. Drying shrinkage values (Figure 3) were calculated using Equation (2).

(2) $$\varepsilon_{t}=\frac{l_{0}-l_{t}}{l}$$

where $\varepsilon_{t}$ is the natural drying shrinkage value of the mortar specimen at *t* days; $l_{0}$ is the initial length of the specimen after forming (including the length of the measuring nails) (mm); $l_{t}$ is the length of the test specimen (with measuring nail) at the age *t* (mm); and l is the length of the specimen without measuring nails. The test result is the arithmetic average of the three specimens, and the final drying shrinkage is accurate to 1.0 × 10^−4^.

## 3. Results

### 3.1. Mechanical Properties

**Figure 4:**
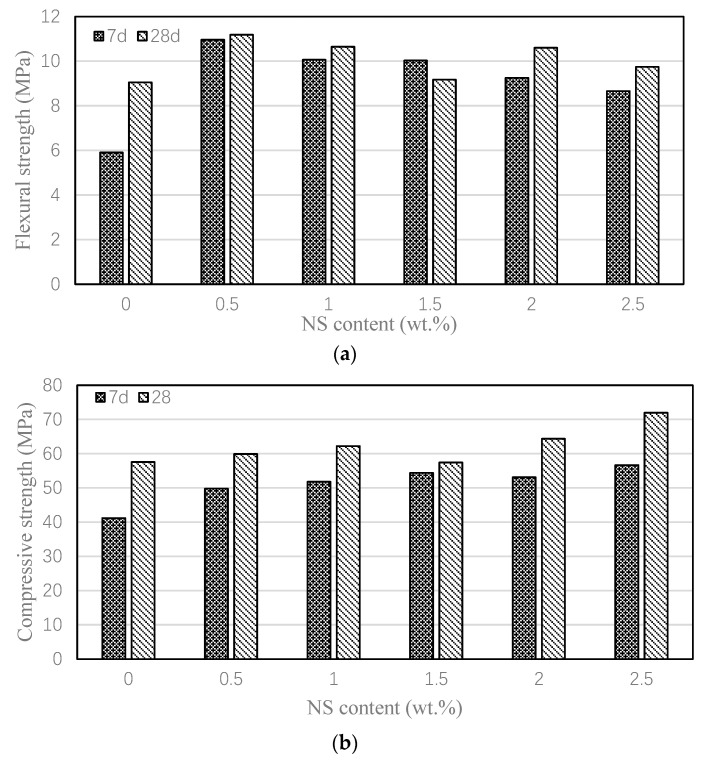
Effect of NS on the flexural and compressive strengths of various mortars. (**a**) Flexural strengths; (**b**) Compressive strengths.

Figure 4 demonstrates the influence of NS on the mortar’s mechanical performance. Figure 4a demonstrates that NS incorporation enhanced the mortar’s 7-day and 28-day flexural strength. For the control mix (FA), the 7-day flexural strength was only 5.9 MPa. After adding 0.5% nano-silica (i.e., NS0.5), the 7-day flexural strength reached 11.0 MPa, which is 85.8% higher than control mortar. The flexural strength increased by 46.6~85.8% for different NS contents, where the mortar with 0.5% NS had the largest flexural strength. Upon reaching the 28-day curing period, the increasement was 1.3~23.5%, in which the mortar with 0.5% NS had the best performance again. Figure 4b presents the compressive strength development of the mortars at 7 and 28 days. The control mortar exhibited compressive strengths of 41.2 MPa at 7 days and 57.6 MPa at 28 days. Upon incorporating 0.5% to 2.5% NS, the 7-day compressive strength improved by 20.9% to 37.4%, while the 28-day strength showed an increase of 0% to 25.3%. These results demonstrate that NS effectively enhances both the flexural and compressive strength of high-volume fly ash mortar, particularly at early ages. This improvement can be attributed to the pozzolanic reactivity of NS, which accelerates the activation of fly ash, thereby promoting early strength development [53].

**Figure 5:**
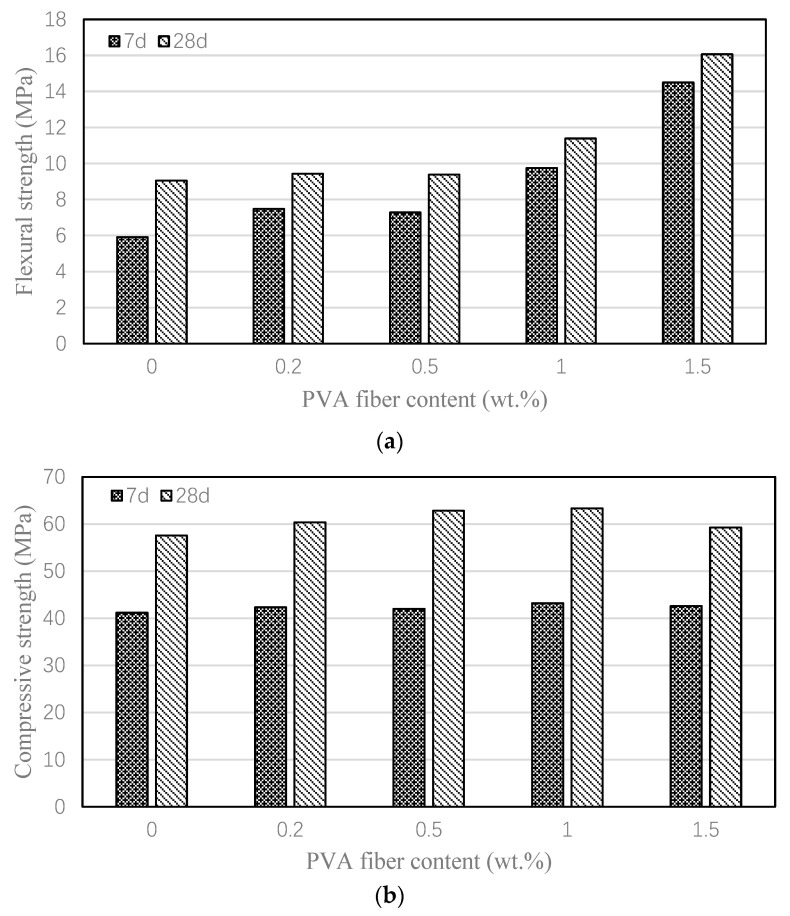
Effect of PVA fiber on the flexural and compressive strengths of various mortars. (**a**) Flexural strengths; (**b**) compressive strengths.

Figure 5 illustrates the influence of PVA fibers on the mechanical properties of high-volume fly ash mortar. As evident from the results, PVA fibers substantially enhanced the flexural strength of the mortar. At an optimal PVA fiber content of 1.5%, the 28-day flexural strength peaked at 16.1 MPa, representing a remarkable 77.6% increase compared to the plain mortar. In contrast, the improvement in compressive strength was relatively modest, with the maximum enhancement (10.0% increase over the control) occurring at a PVA fiber content of 1.0%.

**Figure 6:**
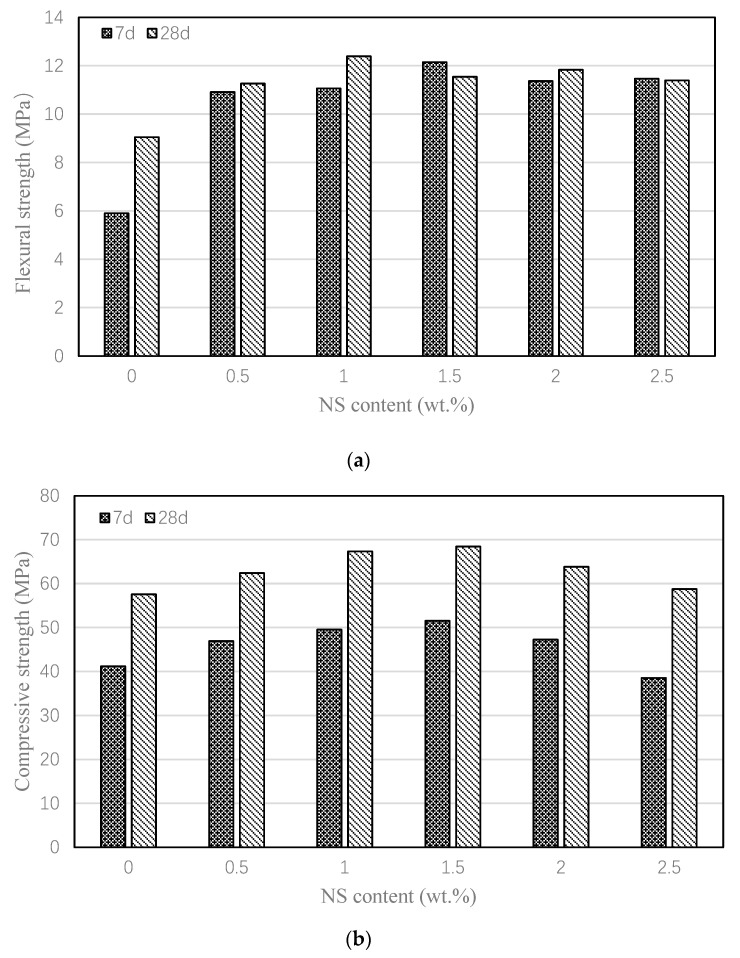
Effect of NS and 1.0%PVA fiber on the flexural (**a**) and compressive strengths (**b**) of various mortars.

Figure 6 demonstrates that the synergistic effect of NS and PVA fibers significantly improves the mechanical properties of mortar. In this experimental series, while maintaining a constant PVA fiber content of 1.0%, NS incorporation initially improved mechanical strengths, but further increases in content ultimately led to diminished performance. The most pronounced enhancement occurred at 7 days, where the modified mortar exhibited maximum increases of 105.8% in flexural strength and 25.1% in compressive strength relative to the control specimen. At 28 days, the optimal NS dosage yielded strength improvements of 36.9% (flexural) and 18.9% (compressive). This performance enhancement stems from two mechanisms: NS promotes pozzolanic reactions, increasing fly ash reactivity and early-age strength development, while PVA fibers provide effective toughening and crack-bridging effects throughout the mortar matrix. Moreover, NS can further boost the reactivity of fly ash by participating in cement hydration reactions, generating substances like hydrated calcium silicate. It can also create physical Van der Waals forces with PVA fiber and establish chemical bonding through hydroxyl groups on the surface of the PVA fiber and dehydrated hydroxyl groups in hydrated calcium silicate [54]. This close connection between the PVA fiber ends and the cementitious matrix enhances the anchorage of the PVA fibers, making them less prone to being pulled out or damaged, thereby bolstering the mechanical properties of the mortar.

### 3.2. Capillary Water Absorption

**Figure 7:**
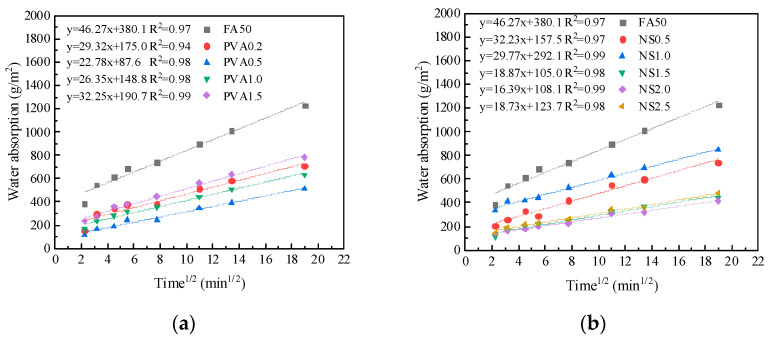
Influence of PVA fiber (**a**) and NS (**b**) on the capillary water absorption of mortar.

**Figure 8:**
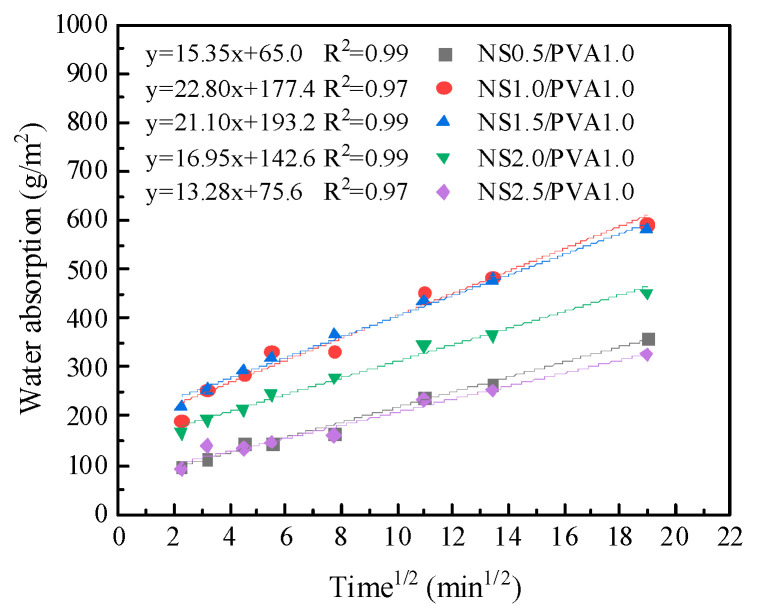
Influence of PVA fiber and NS on the capillary water absorption of mortar.

Figure 7 and Figure 8 show the capillary water absorption test results, where the unit area water absorption is plotted against the square root of time. The slope of the curves is obtained through linear regression, which is also shown in the figures. The slope represents the early capillary absorption rate of cement-based composite materials, as shown in Equation (3).

(3) $$I=k\sqrt{t}$$

where *k* represents the capillary absorption rate; *t* represents the water absorption time in min; and *I* represents the cumulative water absorption of the unit area of the sample at the time *t*.

Figure 7a illustrates the capillary absorption rate of PVA fiber-modified cement-based composite materials as a function of their PVA fiber dosage. It can be observed that the capillary absorption rates of cement mortars including PVA fibers are lower than that of the control mortar. Additionally, the capillary absorption rates show a trend in first decreasing and then increasing with the increase in PVA fiber dosage. When the PVA fiber content is 0.5%, the capillary absorption rate reaches a minimum, which is 50.8% lower (i.e., 22.78 g/(m^2^∙min^1/2^) vs. 46.27 g/(m^2^∙min^1/2^)) than the control mortar. The primary reason is that the addition of fibers can reduce cracking and control cracks. However, when the PVA fiber dosage is too high, uneven dispersion and aggregation may occur, leading to a decrease in the flowability of the cement mortar, an increase in porosity, and consequently an increase in capillary absorption rates.

Figure 7b presents the capillary absorption rates of NS-modified mortars. The results demonstrate that NS incorporation significantly reduces the mortar’s capillary absorption rate. As NS dosage increases, the absorption rate initially decreases before exhibiting a rebound. At the optimal dosage of 2.0%, the absorption rate reaches its minimum value of 16.39 g/(m^2^∙min^1/2^), representing a 62.2% reduction compared to the control mortar. This improvement can be attributed to two mechanisms: (1) NS particles effectively fill matrix micropores, reducing average pore size; and (2) NS activates pozzolanic reactions in fly ash, promoting additional C-S-H gel formation. These synergistic effects densify the mortar microstructure, decrease pore connectivity, and ultimately inhibit capillary water absorption.

Figure 8 presents the capillary absorption rates of mortars incorporating both PVA fibers and NS. The results reveal that the combined use of NS and PVA fibers yields superior water resistance compared to their individual applications. When maintaining a constant PVA fiber content of 1.0%, the capillary absorption progressively decreases with increasing NS content. At the maximum NS dosage of 2.5%, the composite exhibits a remarkable 71.3% reduction in capillary absorption relative to the control mortar. This synergistic effect originates from three mechanisms: (1) NS’s pozzolanic activity enhances cement hydration; (2) NS nanoparticles effectively fill matrix pores, refining the pore structure; and (3) the hydroxyl groups from C-S-H chemically interact with the surface functional groups of PVA fibers, creating a denser interfacial transition zone. These combined effects significantly inhibit capillary water penetration.

### 3.3. Drying Shrinkage

**Figure 9:**
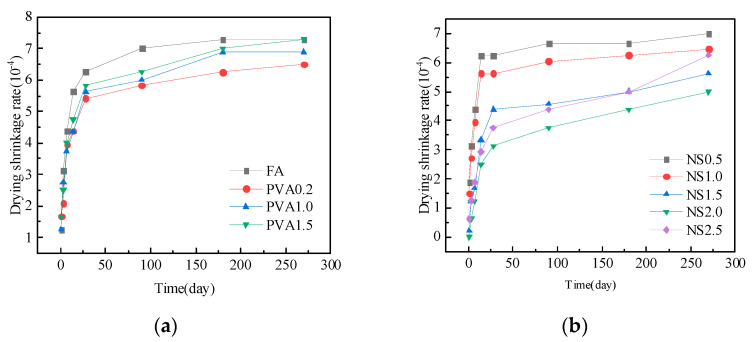
Influence of PVA fiber (**a**) and NS (**b**) on the drying shrinkage of mortar.

Figure 9a illustrates the influence of different PVA fiber contents on the drying shrinkage of fly ash mortar at different ages. As shown in this figure, drying shrinkage increases with the age of the mortar significantly at early ages, while it tends to stabilize at later ages. PVA fiber reinforcement substantially restrained drying shrinkage through microcrack bridging. However, the drying shrinkage of mortar exhibits a gradual increase with higher PVA fiber content. At a 0.2% fiber content, the mortar demonstrates optimal shrinkage resistance, showing a 14.3% reduction in drying shrinkage compared to the control specimen after 180 days of curing. This improvement can be attributed to the bridging effect of PVA fibers, which effectively restrains shrinkage deformation. Nevertheless, when the fiber content exceeds this optimal level, two detrimental effects emerge: (1) fiber dispersion becomes increasingly non-uniform, and (2) the restraining efficiency deteriorates due to fiber clustering. These factors collectively lead to elevated drying shrinkage at higher fiber dosages.

Figure 9b illustrates the effect of NS on mortar drying shrinkage development. The results demonstrate that NS incorporation significantly reduces drying shrinkage across all testing ages. After 180 days of curing, mortars containing 0.5~2.5% NS show 8.5~40.0% lower shrinkage compared to the control specimen. Notably, the shrinkage reduction exhibits a dosage-dependent trend: it progressively decreases with an NS content up to 2.0%, beyond which a slight rebound occurs at a 2.5% NS content. This nonlinear behavior suggests the existence of an optimal NS dosage for shrinkage control.

The extent of concrete drying shrinkage is governed by both the quantity and kinetics of water movement, involving physically adsorbed water in the C-S-H gel and capillary water within the cementitious matrix. Generally, the faster the migration rate and the greater the migration amount of adsorbed water, the faster the drying shrinkage deformation rate and the greater the drying shrinkage value [55]. NS has a pore-filling effect, which refines the pores, reduces the porosity within the mortar, and decreases the content of internal pore water, thus preventing drying shrinkage deformation caused by the migration of capillary water. However, when the NS content is excessive, leading to agglomeration and uneven dispersion, it results in an increase in drying shrinkage.

**Figure 10:**
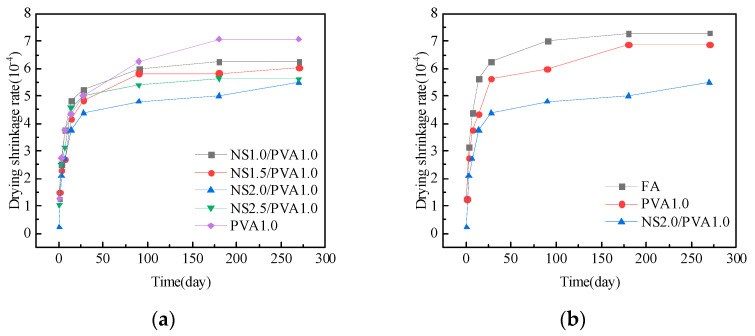
Influence of NS and PVA fiber on the drying shrinkage of mortar. (**a**) PVA alone vs. PVA/NS blend; (**b**) FA vs. PVA1.0 vs. NS2.0/PVA1.0.

Figure 10a presents the development of the drying shrinkage of mortars containing both NS and PVA fiber. Compared to mortars with only PVA fiber, the combined addition of PVA fiber and NS results in a significant reduction in drying shrinkage. Furthermore, with an increase in the content of NS, the drying shrinkage of mortar first decreases and then gradually increases, following a trend similar to that observed when only NS was adopted. When the NS content reaches 2.0%, the mortar exhibits the lowest drying shrinkage, with a 27.3% reduction compared to the group with only PVA fiber at the age of 180 days.

Figure 10b illustrates the drying shrinkage trends in the control mortar, the mortar with 1.0% PVA fiber, and the mortar with a combined addition of 2.0% NS and 1.0% PVA fibers. Compared to the control mortar, the inclusion of 1.0% PVA fiber reduces the drying shrinkage by 5.7% at the age of 180 days. The combined addition of NS and PVA fiber significantly reduces the drying shrinkage of the mortar. At ages of 7, 14, 28, 90, and 180 days, the drying shrinkage is reduced by 38.3%, 33.3%, 30.0%, 31.6%, and 31.4%, respectively, compared to the control mortar.

The synergistic reduction in mortar drying shrinkage through NS and PVA fiber incorporation can be attributed to three primary mechanisms: (1) The bridging effect of PVA fibers effectively inhibits crack initiation, propagation, and connectivity (potential moisture migration pathways), thereby restraining shrinkage deformation. (2) NS nanoparticles function as pore-filling agents, densifying the mortar matrix through physical pore refinement and a consequent reduction in evaporable water content. (3) Chemical interactions between PVA fibers and cement hydrates, particularly through hydrogen bonding with C-S-H gels, modify the gel’s water adsorption behavior and mitigate humidity-induced strain development.

### 3.4. Morphology and Microstructure

**Figure 11:**
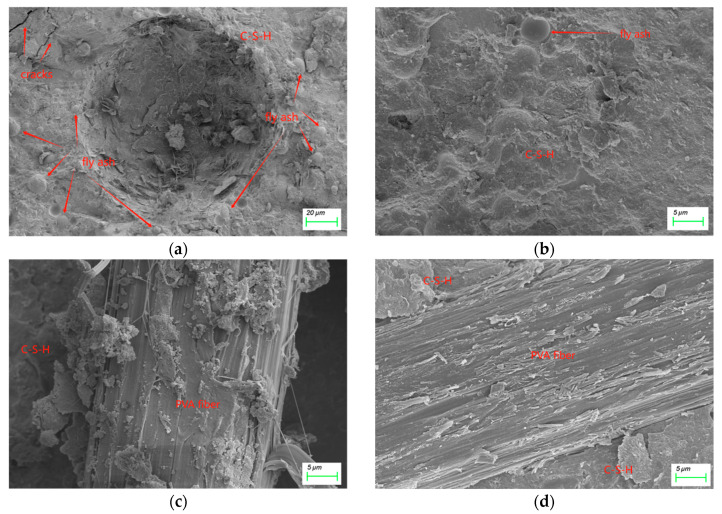
Influence of NS and PVA fiber on microstructures of mortar. (**a**) FA; (**b**) NS2.0; (**c**) PVA1.0; (**d**) NS2.0/PVA1.0.

Figure 11 presents the microstructural characteristics of the mortar specimens. The control mortar (Figure 11a) displays evident microcracks, voids, and numerous unreacted fly ash particles. In contrast, the NS-modified specimen (2.0% NS content, Figure 11b) exhibits a significantly improved microstructure, characterized by enhanced matrix densification with minimal cracks and voids and a substantial reduction in unreacted fly ash particles. This microstructural improvement stems from two synergistic mechanisms of NS: (1) Physical pore-filling effect: the nanoscale particles effectively occupy capillary pores and interfacial transition zones. (2) Chemical activation: the pozzolanic reactivity of NS accelerates cement hydration and promotes secondary C-S-H formation through fly ash activation. These combined effects result in a more homogeneous and denser cementitious matrix, ultimately leading to improved mechanical performance.

Figure 11c shows the internal structure of mortar with a single addition of 1.0% PVA fiber. PVA fibers are bonded to the surrounding cementitious matrix, but they are not well connected to the adjacent bulk of the cementitious matrix. This indicates that when PVA fibers are added alone, they can bond with a limited amount of the cementitious matrix, improving the mortar’s strength and restricting shrinkage changes. However, this bonding is not very strong, and its effect on enhancing mortar strength and limiting shrinkage is not very effective.

Figure 11d presents the internal morphology of a mortar with a combined addition of 2.0% NS and 1.0% PVA fiber. It can be observed that the PVA fibers are closely integrated with the surrounding cementitious matrix, and there is hardly any visible interface between the PVA fibers and the cementitious matrix. They are almost perfectly bonded, allowing the PVA fibers to restrict the formation and development of cracks effectively. This integration significantly enhances mortar strength and effectively reduces shrinkage changes.

**Figure 12:**
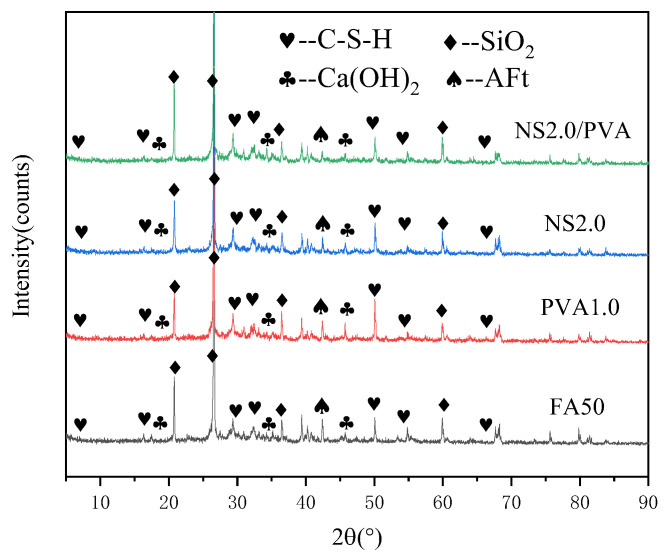
XRD patterns of mortar.

Figure 12 displays the XRD patterns of the mortar specimens, revealing their characteristic hydration products and their relative quantities. Distinct calcium silicate hydrate (C-S-H) diffraction peaks are observed at 2θ angles of 7°, 16°, 29°, 32°, 49°, 54°, 60°, and 66° [56,57]. Notably, the NS2.0 and NS2.0/PVA modified mortars exhibit significantly enhanced peak intensities at 29° and 49° compared to conventional fly ash mortar, demonstrating that NS effectively accelerates the hydration reactions between fly ash and cement, thereby promoting additional C-S-H formation.

The characteristic peaks for portlandite (Ca(OH)_2_) at 18°, 34.1°, and 47.1° show markedly low intensities, indicating substantially reduced portlandite content [58,59]. This depletion is attributed to the pozzolanic reaction of fly ash consuming Ca(OH)_2_ during cement hydration. Remarkably, while the synergistic incorporation of NS and PVA fibers leads to increased C-S-H generation, no corresponding rise in portlandite content is detected. This observation correlates perfectly with the densified microstructure evidenced in the corresponding SEM images, confirming the microstructure–property relationship in the modified mortar systems.

**Figure 13:**
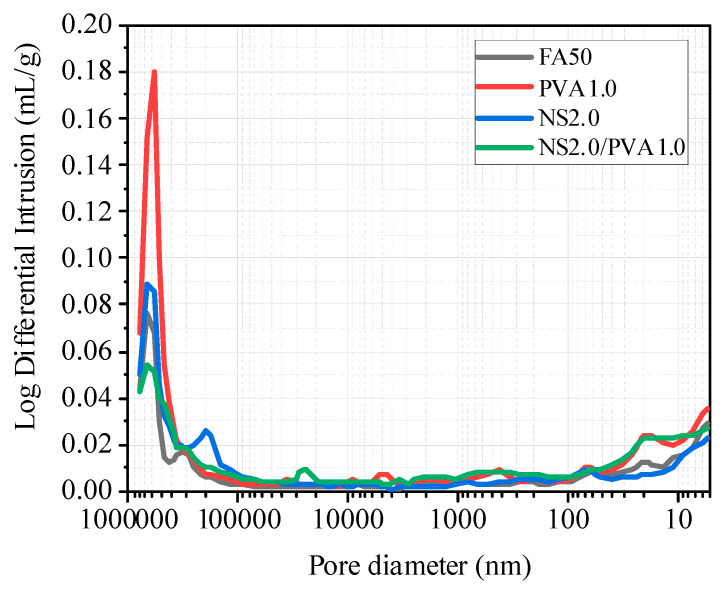
Influence of NS and PVA fiber on pore structures of mortar.

The results of the mercury intrusion test are shown in Figure 13. The pore structure analysis reveals distinct characteristics among different mortar compositions. Plain fly ash mortar exhibits a relatively high volume of macropores. Interestingly, the addition of PVA fibers leads to increased overall porosity and macropore content, with particularly notable growth in the population of detrimental pores exceeding 400 μm in diameter—significantly higher than observed in the reference fly ash mortar. In contrast, NS demonstrates an effective pore refinement capability in fly ash mortar systems. When incorporated into PVA fiber-reinforced mortar, NS produces two notable effects: a substantial reduction in macropore quantity and a marked increase in smaller pores, especially those below 100 nm in diameter. This microstructural modification suggests that NS promotes pore structure refinement through both pozzolanic reaction and physical filling effects. Compared to ordinary fly ash mortar, the NS2.0/PVA1.0 group showed a reduction in macropore quantity and an increase in small pore quantity, effectively improving the internal pore structure of the mortar. Combined with the results from the micro-scanning electron microscope (Figure 11) and the mechanical performance and drying shrinkage tests, the incorporation of NS and PVA fibers significantly enhanced the mechanical properties of fly ash mortar, reduced water absorption, and lowered the drying shrinkage rate.

## 4. Discussion

During the early hydration stage (<7 days), FA primarily functions as a physical filler in cementitious systems. Its spherical particles optimize particle packing density and reduce interparticle friction, thereby improving the workability of fresh concrete. Concurrently, the dissolution of the glassy passive layer on FA particles occurs progressively, preparing for subsequent pozzolanic reactions. In the mid-to-late hydration stages (>7 days), FA reacts with portlandite (Ca(OH)_2_) through pozzolanic reactions to form additional C-S-H gels, which enhance microstructural densification. The incorporation of NS accelerates the depolymerization of the FA glassy network by providing nucleation sites, while reducing the reaction activation energy, thereby significantly promoting the long-term pozzolanic reactivity of FA [20]. The composite addition of NS and PVA fibers effectively enhances the strength of the mortar and reduces the drying shrinkage. The reasons for this are as follows: (1) NS acts as a pore-filling agent, effectively filling the voids in the cement mortar, resulting in a dense structure. (2) NS increases the reactivity of fly ash and promotes the formation of the hydration product C-S-H [60,61,62]. The functional groups on the surface of C-S-H chemically bond with the functional groups on PVA fibers, facilitating a favorable chemical interaction between the PVA fibers and the cementitious matrix (as shown in Figure 11d). (3) The bridging effect of PVA fibers effectively restrains shrinkage variations and crack propagation.

## 5. Conclusions

(1) NS enhances early-age mortar strength, with 0.5% NS increasing 7-day flexural strength by 85.8% and 1.5% NS boosting compressive strength by 32.1%. PVA fibers further improve performance, as a 1.5% content achieves 77.6% higher 28-day flexural strength versus ordinary mortar.
(2) NS and PVA fibers synergistically enhance mortar mechanics. At 7 days, flexural and compressive strengths increase by 105.8% and 25.1%, respectively. By 28 days, the NS-PVA mix maintains improvements of 36.9% (flexural) and 18.9% (compressive) versus plain mortar, demonstrating complementary reinforcement.
(3) NS and PVA fibers significantly reduce the mortar’s capillary absorption. With 0.5% PVA, absorption drops by 50.8%; with 2.0% NS, it decreases by 62.2%. The optimal mix (1.0% PVA + 2.5% NS) reduces absorption by 71.3% compared to ordinary fly ash mortar.
(4) Fly ash mortar exhibits significant early-age drying shrinkage that stabilizes over time. Both NS and PVA fiber independently reduce shrinkage, with 2.0% NS decreasing it by 40.0% and 0.2% PVA fibers by 14.3% at 180 days. The combined use of 2.0% NS and 1.0% PVA fibers shows superior performance, reducing shrinkage by 38.3% (7 d), 33.3% (14 d), 30.0% (28 d), 31.6% (90 d), and 31.4% (180 d) compared to plain mortar. This synergy stems from NS refining pore structure and PVA fibers restraining crack development.
(5) Microstructure analysis shows that NS fills mortar voids, reducing porosity and densifying the structure, while PVA fibers bond tightly with the matrix, inhibiting crack formation. Together, they improve mechanical properties and significantly reduce drying shrinkage.

## Figures and Tables

**Table 1:** Physical and chemical properties of binder.

	SiO_2_/%	Al_2_O_3_/%	CaO/%	MgO/%	Fe_2_O_3_/%	K_2_O/%	MnO/%	SO_4_/%	Specific Surface, Blaine (m^2^/kg)	Compressive Strength, 28-Day (MPa)
Cement	18.3	4.5	62.4	2.1	0.3	1.5	2.3	2.97	350	50.6
Fly ash	46.7	33.8	8.46	-	6.05	1.24	-	1.34	389	-

**Table 2:** Performance specifications of NS.

	Content (%)	Average Diameter (nm)	Specific Surface, Blaine (m^2^/g)	Volume Density (g/cm^3^)	Density (g/cm^3^)
nano-SiO_2_	99.9	20	240	0.06	2.2–2.6

**Table 3:** Basic parameters of PVA fiber.

	Length (mm)	Diameter (mm)	Density (g·cm^−3^)	Tensile Strength (MPa)	Elastic Modulus, (GPa)
PVA fiber	12	0.026	1.3	1560	36.3

**Table 4:** Mix proportions of cement mortars with high-volume of fly ash.

	Mix Proportions (kg/m^3^)				Nano SiO_2_ (wt. %)	PVA Fiber (vol. %)	Superplasticizer (wt. %)
	Water	Cement	Fly ash	Sand			
FA	240	600	600	1200	0.0	0.0	2.0
NS0.5	240	600	600	1200	0.5	0.0	2.0
NS1.0	240	600	600	1200	1.0	0.0	2.0
NS1.5	240	600	600	1200	1.5	0.0	2.0
NS2.0	240	600	600	1200	2.0	0.0	2.0
NS2.5	240	600	600	1200	2.5	0.0	2.0
PVA0.2	240	600	600	1200	0.0	0.2	2.0
PVA 0.5	240	600	600	1200	0.0	0.5	2.0
PVA 1.0	240	600	600	1200	0.0	1.0	2.0
PVA 1.5	240	600	600	1200	0.0	1.5	2.0
NS0.5/PVA 1.0	240	600	600	1200	0.5	1.0	2.0
NS1.0/PVA 1.0	240	600	600	1200	1.0	1.0	2.0
NS1.5/PVA 1.0	240	600	600	1200	1.5	1.0	2.0
NS2.0/PVA 1.0	240	600	600	1200	2.0	1.0	2.0
NS2.5/PVA 1.0	240	600	600	1200	2.5	1.0	2.0

## Data Availability

Data are contained within the article.
